# Alterations of the gut microbiome of largemouth bronze gudgeon (*Coreius guichenoti*) suffering from furunculosis

**DOI:** 10.1038/srep30606

**Published:** 2016-07-28

**Authors:** Tongtong Li, Meng Long, Cheng Ji, Zhixin Shen, François-Joël Gatesoupe, Xujie Zhang, Qianqian Zhang, Lanli Zhang, Yuanli Zhao, Xinhua Liu, Aihua Li

**Affiliations:** 1 State Key Laboratory of Freshwater Ecology and Biotechnology, Institute of Hydrobiology, Chinese Academy of Sciences, Wuhan 430072, China; 2 Key Laboratory of Environmental and Applied Microbiology, CAS; Environmental Microbiology Key Laboratory of Sichuan Province, Chengdu Institute of Biology, Chinese Academy of Sciences, Chengdu 610041, China; 3 Center for Circadian Clocks, School of Basic Medicine & Biological Sciences, Soochow University, Suzhou 215123, China; 4 Qinghai Provincial Fishery Environmental Monitoring Center, Xining 810000, China; 5 INRA, UMR 1419, Nutrition Metabolism and Aquaculture. Ifremer, Centre de Bretagne, 29280 Plouzané, France

## Abstract

High-throughput sequencing was applied to compare the intestinal microbiota in largemouth bronze gudgeon either healthy or affected by furunculosis. Proteobacteria, Actinobacteria, Tenericutes, Firmicutes and Bacteroidetes were detected as the predominant bacterial phyla in the gut of both diseased and healthy fish. The abundance of Proteobacteria differed significantly between the two groups of fish, mainly due to the overwhelming prevalence of *Aeromonas* in the diseased fish (81% ± 17%), while the genus was unevenly spread among the apparently healthy fish (33% ± 33%). The bacterial diversity in the intestine of diseased fish was markedly lower than in healthy fish. Analysis revealed the significant dissimilarity between the gut microbiota of diseased and healthy fish. The bacterial profiles in the gut were further characterized with the 28 phylotypes that were shared by the two groups. In diseased fish, two shared OTUs (OTU0001 and OTU0013) were closely related to *Aeromonas salmonicida*, their total proportion exceeding 70% of the sequences in diseased fish, while averaging 5.2% ± 4.6% in the healthy fish. This result suggested the presence of healthy carriers of pathogenic *A. salmonicida* among the farmed fish, and the gut appeared as a probable infection source for furunculosis in largemouth bronze gudgeon.

Gut microbiota can play important roles in nutrition and health, and it may be considered as an integral component of the host^1^,^2^,^3^,^4^. Teleosts are in direct contact with the aquatic environment, and thus in continual contact with complex and dynamic microbiota, possibly interacting with health^5^. In addition, gut microbiota may harbor opportunistic pathogens, indicating that the gastrointestinal tract is a potential pathway for pathogen invasion^6^,^7^. Recent studies have revealed the important contributions of gut microbiota to vertebrate health and disease, stimulating the interest in understanding how gut microbial communities are assembled and how they impact host fitness^8^. Such studies have been increasingly carried out on fish gastrointestinal microbiota. Most data concerned healthy fish, with a view to investigate the factors that may contribute to shape gastrointestinal microbiota, such as diet, behavior and genotype of the host^7^,^9^,^10^,^11^,^12^. Few studies have yet focused on the interaction between gut microbiota and disease in fish by using next generation sequencing^13^.

Largemouth bronze gudgeon (*Coreius guichenoti*), belonging to the family Cyprinidae and the order Cypriniformes, is an endemic and economically important species, distributed only in the middle and upper reaches of Yangtze River^13^,^14^. The population of largemouth bronze gudgeon has significantly declined because of environmental pollution, overexploitation, and the construction of electric power plants in this area. The aquaculture of this species began in 2005 for supplying the natural population, and for commercial exploitation. However, diseases have emerged with the development of intensive culture.

Most *Aeromonas* species include opportunistic pathogens, which can infect wounded or stressed and immunocompromised hosts^15^. Historically, *Aeromonas salmonicida* has been recognized as the most important bacterial salmonid pathogen because of its severe economic impact on aquaculture^16^. Translocation of bacteria through the foregut was identified among the likeliest infection routes of *A. salmonicida* in Atlantic salmon^17^,^18^. Other studies have reported that this bacterium could also infect non-salmonid fishes^19^,^20^,^21^,^22^. This infection has been also diagnosed in largemouth bronze gudgeon (unpublished data), and it might cause the disease outbreaks that decimated the natural population. The prevention of this widespread disease was difficult due to the lack of knowledge about the infection sources and routes, and further investigation in this area is of the utmost importance.

Gut is considered to be an important infection channel of many diseases, and enteritis is a common symptom of furunculosis. The importance of the intestinal tract as infection source in fish was previously evidenced in the case of motile *Aeromonas* septicemia, as the pathogenic clone of *Aeromonas hydrophila* that caused the disease was detected in the intestine before systemic infection^23^.

The present experiment focused on the emergence of furunculosis in largemouth bronze gudgeon cultured in net-cages from the perspective of intestinal microbial ecology. The first issue was to compare the relative abundances of gut bacteria in diseased and healthy fish, in view to evaluate the changes in microbiota that may be caused by the disease. To this end, intestinal contents of diseased and healthy fish were sampled in the same farming unit, and analyzed by high throughput sequencing. The second purpose of this study was to investigate the hypothesis that furunculosis may occur in the same way as motile *Aeromonas* septicemia. In other words, a particular attention was paid to the detection of the phylotypes of pathogenic *Aeromonas salmonicida* in the gut of healthy fish, as a possible prerequisite for furunculosis outbreak. In addition, zebrafish were challenged with bacterial isolates corresponding to the pathogenic phylotypes to assess virulence, and to check the reproducibility of furunculosis symptoms.

## Results

### Sequencing depth and alpha diversity indices

After initial quality filtering, removal of chimeras and “contaminants” sequences, 3,692 to 26,518 effective sequences were collected from each sample, resulting in a final dataset of 161,417 high quality sequences from the eleven samples. The average read length was 170 bp. For the downstream alpha and beta diversity analyses, the sequence number was normalized at 3,692 by randomly subsampling to standardized sampling effort. The subsampling of the sequences retained a sufficient resolution to compare the bacterial communities, as suggested by an average Good’s coverage of 97.44% ± 1.28% (mean ± SD, Table 1) and by the rarefaction curves (Figure S1). After sample-size standardization, 40,612 sequences remained, which clustered into a total of 1,184 OTUs at the 97%-similarity level. Each sample contained 49 to 328 OTUs (Table 1). The microbial complexity in the gut was estimated using alpha-diversity indices of the taxonomic profiles at the 97%-similarity threshold (Table 1).

The bacterial community diversity was measured by both Shannon and Simpson indices. A significantly greater diversity of bacterial community was found in the samples from healthy fish, compared to those from diseased fish (F = 9.7, *p* < 0.05 and F = 11.7, *p* < 0.05, respectively). In healthy fish, the total number of OTUs and the richness estimator Chao1 tended to be higher than those in diseased fish, but not significantly (F = 2.8, *p* > 0.05 and F = 0.5, *p* > 0.05, respectively).

### OTU-based beta diversity of the intestinal microbial communities

**Figure 1:**
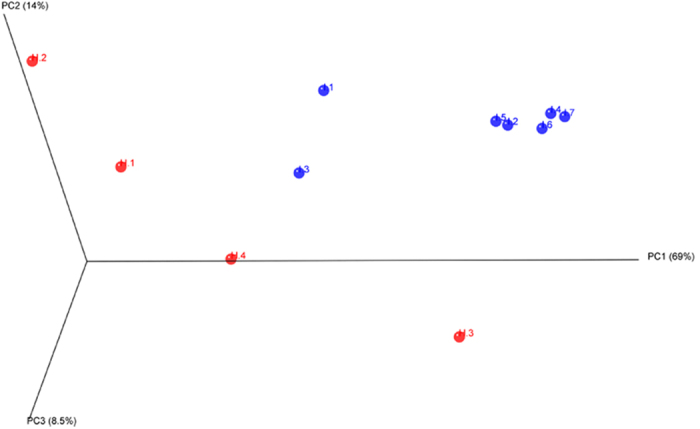
Three-dimensional principal coordinate analysis of the community structure using weighted UniFrac distances. H.1-H.4 stand for the intestinal samples of healthy fish, and I.1-I.7, for the intestinal samples of diseased fish. The distances between symbols on the ordination plot reflect the relative dissimilarities in community structures.

The gut microbial community structures of healthy and diseased fish were compared using the Principal Coordinate Analysis (PCoA), based on weighted UniFrac distance matrixes. PCoA plot showed that the bacterial communities clustered tightly among the samples from diseased fish, remaining distinct from the cluster corresponding to the healthy fish along PC1 (69% variation explained, Fig. 1). The two groups of bacterial community were significantly different according to the UniFrac *P*-test. The normalized weighted UniFrac distances among the individuals ranged from 0.04 to 0.56 (average: 0.29) for the diseased fish and from 0.35 to 0.88 (average: 0.58) for the healthy fish (F = 14.1, *p* < 0.05).

### Taxonomic profiling of the bacterial community

**Figure 2:**
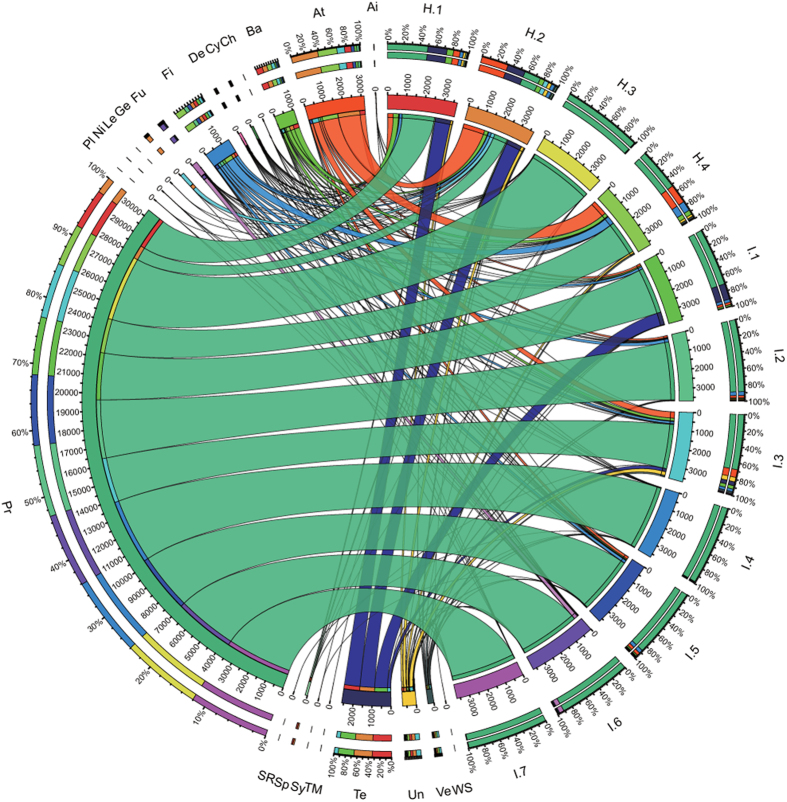
Circular representation of the intestinal microbial communities of C. guichenoti in both groups at the phylum level. The inner circular diagram shows the relative abundance of different phyla in fish gut samples; the sequences that could not be assigned at the phylum level were designated as ‘unclassified bacteria’; H.1-H.4 stand for the intestinal samples of healthy fish, and I.1-I.7, for the intestinal samples of diseased fish. Abbreviations: Ai-Acidobacteria, At-Actinobacteria, Ba-Bacteroidetes, Ch-Chloroflexi, Cy-Cyanobacteria_Chloroplast, De-Deinococcus-Thermus, Fi-Firmicutes, Fu-Fusobacteria, Ge-Gemmatimonadetes, Le-Lentisphaerae, Ni-Nitrospira, Pl-Planctomycetes, Pr-Proteobacteria,Sp-Spirochaetes, SR-SR1,Sy-Synergistetes, Te-Tenericutes, TM-TM7,Un-unclassified bacteria, Ve-Verrucomicrobia and WS-WS3.

The distribution of OTUs at the phylum level in healthy and diseased fish was illustrated in Fig. 2. The phylogenetic classification of the sequences resulted in the identification of 21 phyla (18 and 20 phyla detected in the diseased and healthy fish, respectively). The sequences that could not be classified into any known group were assigned as “unclassified bacteria”.

Proteobacteria dominated the intestinal microbial community of *C. guichenoti* in both groups (Fig. 2). Compared with the healthy fish, the diseased fish had a significantly higher level of Proteobacteria (diseased: 86% ± 12%; healthy: 55% ± 33%). The next most abundant phyla (average relative abundance >1%) were Actinobacteria (diseased: 2.9% ± 3.3%; healthy: 16.3% ± 14.8%), Tenericutes (diseased: 3.5% ± 7.3%; healthy: 11.5% ± 13.3%), Firmicutes (diseased: 2.6% ± 1.9%; healthy: 5.2% ± 5.1%) and Bacteroidetes (diseased: 1.3% ± 1.5%; healthy: 4.9% ± 3.4%). Unclassified bacteria were particularly abundant in the gut microbiota of healthy fish, but the difference between groups was not significant (diseased: 1.4% ± 1.9%; healthy: 2.6% ± 1.8%, two-tailed Student’s *t*-test, *p* > 0.05). Other phyla were recurrently detected in the intestinal microbial community of *C. guichenoti* (average relative abundance >0.1%), namely: Verrucomicrobia (diseased: 0.60% ± 0.81%; healthy: 1.11% ± 0.91%), Fusobacteria (diseased: 1.02% ± 2.11%; healthy: 0.18% ± 0.03%), Gemmatimonadetes (diseased: 0.00% ± 0.01%; healthy: 1.35% ± 2.67%), Deinococcus-Thermus (diseased: 0.21% ± 0.26%; healthy: 0.53% ± 0.41%), Spirochaetes (diseased: 0.02% ± 0.04%; healthy: 0.79% ± 0.88%) and Cyanobacteria_Chloroplast (diseased: 0.27% ± 0.31%; healthy: 0.33% ± 0.01%). Few phyla occurred at low abundance and sporadically in some intestinal samples (in total: approximately 0.13% of the sequences; namely: Acidobacteria, Nitrospira, Chloroflexi, TM7, Lentisphaerae, Synergistetes, Planctomycetes, WS3 and SR1).

**Figure 3:**
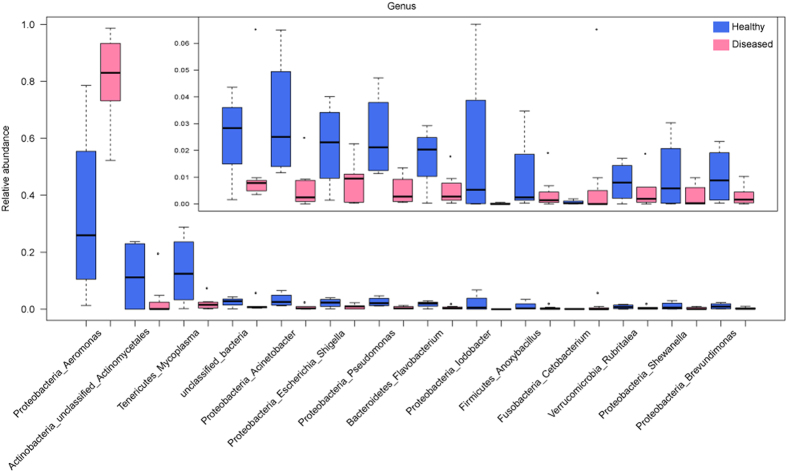
Differentially predominant genera (relative abundance >0.5%) in healthy and diseased fishes. The blue and red boxes represent healthy and diseased fishes, respectively. The boxes include the interquartile range (IQR), from the first and third quartiles, and the inside bold line represents the median. The dotted lines extending vertically from the boxes (whiskers) denote the lowest and highest values within 1.5 IQR from the first and third quartiles. The circles represent outliers beyond the whiskers.

At the genus level, *Aeromonas* was the dominant phylotype in both groups. Its abundance averaged 33% ± 33% in the healthy fish, but it was significantly higher in the diseased fish (81% ± 17%; Fig. 3; F = 10.7 and *p* < 0.05). Except *Cetobacterium*, which was more abundant in the diseased fish, the abundance of the other genera and unclassified taxonomic groups was higher in the healthy fish (unclassified_Actinomycetales, *Mycoplasma*, *Acinetobacter*, unclassified_bacteria, *Pseudomonas*, *Escherichia*_*Shigella*, *Iodobacter*, *Flavobacterium*, *Shewanella*, *Brevundimonas*, *Anoxybacillus* and *Rubritalea*; Fig. 3).

**Figure 4:**
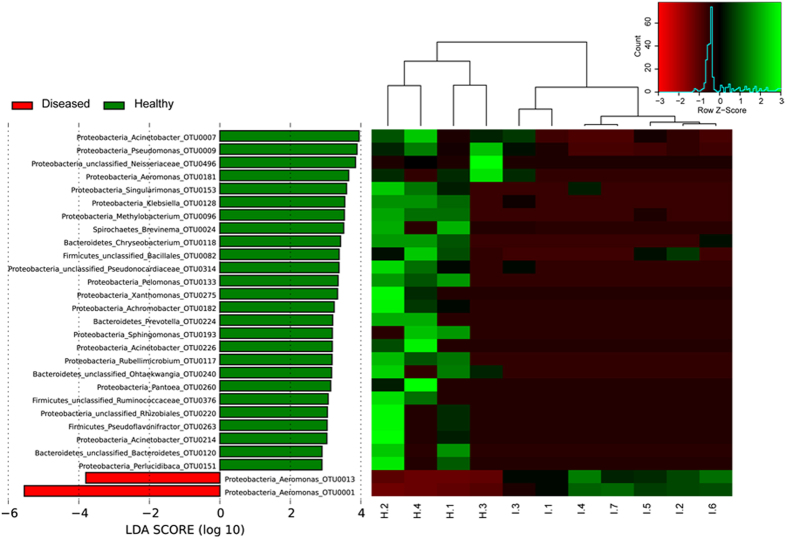
LEfSe identified the most differentially abundant OTUs (relative abundance >0.01%) between the healthy (green) and diseased (red) fish. The left histogram shows the linear discriminant analysis (LDA) scores computed for the most discriminating OTUs, based on their relative abundance in the gut microbiota of *C. guichenoti*. The right heatmap shows the scores of these relative abundances; H.1-H.4 stand for the intestinal samples of healthy fish, and I.1-I.7, for the intestinal samples of diseased fish.

The most abundant OTUs in both of the healthy and diseased fish belonged to the genus *Aeromonas*. The OTUs with significant difference in abundance between healthy and diseased fish were revealed by LEfSe on the 226 top OTUs (average relative abundance >0.01%). This threshold allowed to keep as many OTUs as possible for meaningful comparisons, while excluding the rarest OTUs. The mean abundance of 28 OTUs was found significantly different between the two groups of samples (Fig. 4). A total of 26 OTUs was significantly more abundant in the healthy fish, including among the most prominent *Acinetobacter*_OTU0007, *Pseudomonas*_OTU0009, and *Brevinema*_OTU0024. Conversely, only two OTUs were overrepresented in the diseased fish, *Aeromonas*_OTU0001 and *Aeromonas*_OTU0013, suggesting that these two OTUs might play an important role in furunculosis.

### Partition between shared and unique OTUs

The taxonomic profiles of the intestinal microbial community of *C. guichenoti* were further analyzed by distinguishing the OTUs that were shared by both groups from the unique representatives (Figure S2). Globally, 250 OTUs were shared among the intestinal samples of healthy and diseased fish, corresponding to 93% and 97% of the total reads in the two groups, respectively. The most abundant OTUs that represented more than 0.5% of the total from at least one group were further considered. Among these OTUs, 25 accounted in total for 76 and 91% of the sequences from healthy and diseased fish, respectively. The phylogenetic affiliation of these OTUs was shown in Table S1. Five of these OTUs (00001, 00002, 00004, 000011 and 00013) were assigned to the genus *Aeromonas*, which dominated the bacterial communities in healthy and diseased fish and they accounted for 33 and 81% of the sequences from the groups, respectively (Table S1). A phylogenetic tree was constructed based on representative sequences of these five OTUs assigned to *Aeromonas* by neighbor-joining (MEGA program version 6.0; Figure S3). Two of these OTUs (00001 and 00013) were clustered with *A. salmonicida*, and remained distinct from the other reference strains (*A. hydrophila*, *A. sobria*, and *A. veronii*), and from the other OTUs that corresponded to same genus (00002, 00004 and 000011). Interestingly, two reference pathogenic strains of *A. salmonicida* were isolated and identified in samples from *C. guichenoti* affected by furunculosis (strains YTU1 and YTL1, isolated from ulcer and liver, respectively).

In an additional experiment, pathogenicity of YTU1 and YTL1 strains were studied using zebrafish as infection model, and the LD_50_ values were determined to be 1.6 × 10^4^ and 1.4 × 10^4^ cfu/fish after intraperitoneal injection, respectively. The clinical signs were similar to the typical signs of furunculosis including swellings and ulcerations in the dorsal muscle, a few blood clots in the back, and a reddened and swollen anus. Pure *A. salmonicida* cultures were obtained from the ulceration, liver, and kidney of moribund zebrafish after the challenge. No control fish developed clinical signs or died.

The proportion of the unique OTUs that were detected only in one group was similar in both groups (67 and 63% of the sequences from healthy and diseased fish, respectively). In terms of abundance, however, the unique OTUs represented a small proportion of the total (7.3 and 2.7% of the sequences from healthy and diseased fish, respectively). The five most abundant representatives of the OTUs that were detected only in healthy fish were affiliated to *Bacteroides*, unclassified_Bacteroidetes, unclassified_Porphyromonadaceae (Bacteroidetes), and *Rubellimicrobium* and *Perlucidibaca* (Proteobacteria; Table S2). The top five unique OTUs in diseased fish corresponded to Cyanobacteria Chloroplast GpI, *Gemmiger*, *Aeromonas* and *Glaciecola* (Proteobacteria), and *Staphylococcus* (Firmicutes; Table S3).

## Discussion

The causative agent of furunculosis, *A. salmonicida* has threatened fish farms for more than a century, and the epidemics induced significant economic losses in the aquaculture industry throughout the world^16^,^24^,^25^,^26^. As a major aquaculture country, China accounts for nearly 70% of the world’s total aquaculture production^27^. The understanding of pathogenic bacteria in the intestine of fish has also increased in the last two decades. Investigations have demonstrated that the intestine is likely to be the main port of entry for several fish pathogens under experimental infections, including *A*. *salmonicida*^17^,^18^,^23^. Numerous studies have successfully used molecular techniques, alone or in combination with conventional methods, to characterize uncultured and culturable bacteria in the gut of a wide range of fish species. The observation of intestinal cellular damages is one of the most common symptoms in fishes affected by furunculosis^28^,^29^, but it is unclear whether the intestinal cellular damages are the cause or the result of the infection by *A. salmonicida*, and that warrants further investigation.

In mice and humans, many gastrointestinal diseases are associated with shifts in the gut microbial community^30^,^31^,^32^,^33^. The study of the association between diseases and alterations in the diversity of intestinal microbiota is essential for understanding how gut microbes may impact host fitness, and for developing efficient measures to prevent or treat the diseases. Thus far, the high-throughput sequencing-based studies that regarded the association of fish intestinal bacterial communities with disease occurrence have been relatively limited. To our knowledge, the present study was the first one that addressed the relationship between furunculosis and fish gut bacterial communities of largemouth bronze gudgeon, an economically significant species in Chinese aquaculture. We found that alpha-diversity was markedly reduced in the gut microbiota of diseased fish. Moreover, the weighted UniFrac distance-based PCoA and *P*-test analysis revealed the significant dissimilarity between the intestinal bacterial community structures of diseased and healthy fish. Interestingly, the normalized weighted UniFrac distances among the samples from diseased individuals were significantly shorter, compared to those from healthy fish. In other words, the bacterial community associated with the intestine of healthy individuals was more diverse than that of diseased fish. It must be kept in mind that the present data concerned the relative abundance of taxonomic units. In diseased fish, the likely overgrowth of the opportunistic bacterium diminished the relative importance of the other taxons, but not necessarily their population density. The preservation of the “microbial balance” in the intestine is critical for health, and everything must be done to inhibit the growth of opportunistic strains of *A. salmonicida* in the gastrointestinal tract of fish, as well as to endeavor to restore microbial diversity after infectious episodes and antibacterial treatments.

In this study, *Aeromonas* was the dominant genus in both of the healthy and diseased fish, but in greater proportion in the latter group. Previous studies revealed that the strains of *Aeromonas* with high-adhesive capability could colonize the surface of intestinal mucosa, and the intestine might be a primary site for stress-induced infections by pathogenic strains^34^.This was in accordance with another study that suggested that fish gut may be a reservoir for many opportunistic pathogens^35^. However, members of the genus *Aeromonas* are normal components of fish gut microbiota^6^,^7^,^12^,^36^,^37^, and the bacteria associated with mucosa may be regarded as indigenous species possibly beneficial to host nutrition, mucosal defense, and host immunity^38^. In the diseased fish, two OTUs, OTU0001 and OTU0013, were closely related to pathogenic strains of *A. salmonicida*, especially YTU1 and YTL1 (Figure S3). These latter strains were isolated from ulcer and liver in *C. guichenoti* affected by furunculosis, and their virulence was demonstrated by the experimental infection of zebrafish (and also grass carp, unpublished data), suggesting that OTU0001 and OTU0013 might play an important role in furunculosis. The total proportion of these two OTUs exceeded 70% of total sequences in the diseased group, but the two OTUs were shared by healthy individuals, averaging 5% of the sequences from the healthy group. This result suggested that pathogenic strains of *A. salmonicida* might be present in the intestine before furunculosis occurred, which is in accordance with a former study about *Aeromonas hydrophila*^23^. Environmental stressors, such as hypoxia, pollutants, or sudden changes in temperature, can weaken the host’s immune system and allow the pathogens to cause diseases by colonizing or invading the intestinal mucosa^39^. Previous study revealed that the “rare biosphere” might simply result from past ecological changes, but rare taxons can become dominant in response to shifts in environmental conditions, when local or global changes favor their growth^40^. The disruption of the ecological equilibrium in the intestine of animals can lead to the overgrowth of pathogenic intestinal bacteria^41^. Stress can alter the intestinal function in fish^42^; and then pathogenic *A. salmonicida* may attack membrane surfaces in fish intestine, translocate across the intestinal wall and propagate the disease. Therefore, dysbiosis and the transformation of microecological niches in fish intestine in response to environmental stresses may cause the occurrence of the disease. This phenomenon highlights the need for manipulating the gut microbiota in fish, with suitable strategies for better health management. The present study may provide some cues to determine the etiology of furunculosis, and to work out effective prevention and control measures.

## Methods

### Sample collection and DNA extraction

A severe disease outbreak of cage-cultured *C. guichenoti* occurred in the upper reaches of the Yangtze River in Luzhou, Sichuan province, Southwest China in late March 2012. The cumulative mortality was approximately 42% during the epidemic at a water temperature ranging from 15 °C to 20 °C. The fish were 18 cm to 20 cm in length. The major symptoms of the diseased fish were swollen dorsal muscle, shallow ulcerations, swollen mouth, slight exophthalmia, and slight hemorrhage on the body surface. The causative agent was identified as *Aeromonas salmonicida* subspecies *salmonicida* by conventional bacteriological methods and molecular sequence data (data not shown; GenBank identifiers: KC254647, KC254648). The fish studied in the present study were reared in net cages (3 m × 3 m × 3 m) in the upper reaches of the Yangtze River. Seven fish showing typical clinical signs and four healthy fish were collected, placed in an ice box, and sent to the laboratory on the same day. The healthy fish were active and feeding normally, and the pathogen was not detected by bacteriological culture from the liver. These healthy fish were sampled from the same population as the diseased fish, and every fish was reared under the same conditions. The methods used in this study were reviewed and approved by the ethics committee of the Institute of Hydrobiology, Chinese Academy of Sciences, and were carried out in accordance with the relevant guidelines, including any relevant details.

Prior to dissection for sample collection, all fish were euthanized with an overdose of MS 222 (Sigma, Germany). All procedures for handling and euthanasia of fish were done according to the procedure described by Roeselers, *et al*.^6^. The total of eleven intestinal samples comprised the four ones from healthy fish (H.1-H.4) and the other seven from diseased fish (I.1-I.7). For every sample, 100 mg of excised intestines were placed into a sterile 2-mL tube with 200 mg of 0.1-mm glass beads, 300 μL lysis solution, and 10 μL Proteinase-K solution (OMEGA, Bio-Tek, USA). The samples were homogenized for 3 min in a bead beater (Biospec Products) and centrifuged at 4,000 × g for 5 min to eliminate the pellet of debris. The supernatant was collected for isolating metagenomic DNA with E.Z.N.A. Stool DNA kit (OMEGA, Bio-Tek, USA) according to the manufacturer’s instructions. The DNA concentration was quantified with NanoDrop ND-1000 (Thermo Scientific, USA). The samples were extracted in duplicates, and the two extracts from the same sample were pooled together to avoid bias^7^,^12^. The extracted DNA was stored at −80 °C until use.

### PCR amplification and sequencing

Universal primer 338F (5′-ACTCCTACGGGAGGCAGCAG-3′) and 533R (5′-TTACCGCGGCTGCTGGCAC-3′) with 6 nt unique barcode at 5′-end of 338F was used to amplify the V3 hypervariable region of 16S rRNA gene^43^. The amplification reaction mix (final volume 25 μL) consisted of 0.25 Unit of TaKaRa Ex Taq DNA polymerase, 2 μL 10 × Ex Taq buffer (Mg2+), 1.6 μL dNTP mix (TaKaRa Biotechnology Co., Ltd., Dalian, China), forward and reverse primers (1.0 μM each), 1 μL BSA (10 mg mL^−1^), 1 μL DNA template (c. 10 ng), and sterile water added up to 25 μL. The amplification process comprised an initial denaturation step of 94 °C for 5 min, followed by 30 cycles at 94 °C for 30 s, 56 °C for 30 s, and 72 °C for 30 s, and a final extension at 72 °C for 10 min. The PCR reactions were duplicated for each sample, and the two products were pooled before migration on 1% agarose gel electrophoresis. The PCR products were re-extracted from the gel, and purified by using the QIAEX II Gel Extraction Kit (QIAGEN). All the samples were sequenced in one paired-end MiSeq run (Illumina Inc., San Diego, CA).

### Statistical and bioinformatics analysis

The sequence data were processed using QIIME Pipeline-Version 1.7.0 (http://qiime.org/)^44^. The sequences with an average phred score lower than 20, ambiguous bases, homopolymer runs exceeding 8 bp, primer mismatches, or sequence lengths shorter than 150 bp were removed. Only the sequences with an overlap longer than 10 bp and without any mismatch were assembled according to their overlap sequence. The reads that could not be assembled were discarded. All the sequence reads were trimmed and assigned to each sample, based on their barcodes. The barcode and sequencing primers were trimmed from the assembled sequence, and aligned with the Bacterial SILVA database (SILVA SSU 123), resulting in 14,956 bacterial sequences^45^. The chimeric sequences were excluded by using the Uchime algorithm^46^,^47^. Taxonomy was assigned using the Ribosomal Database Project classifier, with a confidence threshold of 50%^48^,^49^. The “contaminants” sequences classified as “Mitochondria,” “Eukaryota,” or “unknown,” as well as those from archaeal and eukaryotic 16S/18S rRNA genes were removed to focus as closely as possible on bacterial diversity. The remaining sequences were clustered into operational taxonomic units (OTUs) at 97% identity threshold. All the samples were randomly resampled to 3,692 reads. The rarefaction curves were generated from the remaining numbers of OTUs, before analyzing both alpha-diversity (phylogenetic distance on whole tree; number of OTUs; Chao1 estimator of richness; Shannon and Simpson diversity indices) and beta-diversity with Principal Coordinates Analysis (PCoA) and UniFrac. The microbial communities were visualized using Circos^50^. A phylogenetic tree was generated with MEGA program (version 6.0). The heatmap was constructed using the heatmap 2 function of the R gplots package^51^. The bacterial communities were compared by *p* test on the Fast UniFrac metric matrix and PCoA, based on phylogenetic information^52^. LEfSe (http://huttenhower.sph.harvard.edu/galaxy/) was also utilized, the significance threshold of the alpha parameter for the Kruskal-Wallis test among classes was set to 0.05 and the cut-off logarithmic LDA score was 2.0^53^. Differences between two independent groups were evaluated by Analysis of Variance (ANOVA) (SPSS, version 19.0). The raw sequences were deposited at NCBI/EBI/DDBJ Sequence Read Archive (Accession No. DRA004067).

### Experimental infection

*C. guichenoti* has not been acclimatized well for artificial culture, resulting in difficulty in laboratory trials, particularly for artificial challenge experiments. Thus, we used zebrafish (mean weight: 1.0 ± 0.2 g) as an alternative. The fish were randomly distributed between nine experimental groups of 20 fish each, and reared in 10-L aquaria filled with 5 L of stagnant water at 18 °C for 15 days prior to challenge. Half of the water was exchanged once a day. YTU1 and YTL1 was cultured on TSA plates at 18 °C overnight and suspended in PBS buffer. Zebrafish were intraperitoneally injected with 0.05 mL of four 10-fold serial dilutions of YTU1 or YTL1 suspension at final concentrations ranging from 8 × 10^5^ to 8 × 10^8^ colony-forming units (cfu) per milliliter. Sterile PBS was injected to the control group in the ninth and last aquarium. Clinical signs and mortalities were recorded daily for 15 d post-challenge. Dead fish specimens were subjected to standard microbiological and pathological examinations, and LD_50_ values were calculated according to the methodology of Reed^54^.

## Additional Information

**How to cite this article**: Li, T. *et al*. Alterations of the gut microbiome of largemouth bronze gudgeon (*Coreius guichenoti*) suffering from furunculosis. *Sci. Rep.* **6**, 30606; doi: 10.1038/srep30606 (2016).

## Supplementary Material

Supplementary Information

## Figures and Tables

**Table 1:** Number of sequences analyzed, observed diversity richness (OTUs), estimated OTU richness (Chao1), diversity index (Shannon and Simpson), and estimated sample coverage for 16S rRNA libraries of the different samples.

Samples	OTUs	Chao1	Shannon	Simpson	Coverage (%)
H.1	311	544	3.26	0.11	95.79
H.2	287	402	3.23	0.14	96.98
H.3	76	242	1.85	0.25	98.68
H.4	324	434	4.01	0.06	96.80
I.1	147	385	1.82	0.36	97.57
I.2	162	400	1.20	0.67	97.33
I.3	328	662	3.01	0.19	94.96
I.4	63	201	0.29	0.92	98.78
I.5	166	341	1.31	0.64	97.42
I.6	84	229	0.76	0.75	98.50
I.7	49	165	0.20	0.95	98.99

H.1-H.4 stand for the intestinal samples of healthy fish, and I.1-I.7, for the intestinal samples of diseased fish.
