# Supplementary material for: Alterations of the gut microbiome of largemouth bronze gudgeon (Coreius guichenoti) suffering from furunculosis
Source: Sci Rep. 2016 Jul 28;6:30606. doi: 10.1038/srep30606 (PMC4964610; doi:10.1038/srep30606)
Supplement: Supplementary Information [file srep30606-s1.pdf]

Alterations of the gut microbiome of largemouth bronze gudgeon (*Coreius guichenoti*) suffering from furunculosis

Tongtong Li[1,2*], Meng Long[1*], Cheng Ji[3], Zhixin Shen[4], François-Joël Gatesoupe[5], Xujie Zhang[1], Qianqian Zhang[1], Lanli Zhang[1], Yuanli Zhao[1], Xinhua Liu[1], Aihua Li[1]

[1]State Key Laboratory of Freshwater Ecology and Biotechnology, Institute of Hydrobiology, Chinese Academy of Sciences, Wuhan 430072, PR China

[2]Key Laboratory of Environmental and Applied Microbiology, CAS; Environmental Microbiology Key Laboratory of Sichuan Province, Chengdu Institute of Biology, Chinese Academy of Sciences, Sichuan 610041, PR China

[3]Center for Circadian Clocks, School of Basic Medicine & Biological Sciences, Soochow University, Suzhou 215123, PR China

[4]Qinghai Provincial Fishery Environmental Monitoring Center, Xining 810000, China

[5]INRA, UMR 1419, Nutrition Metabolism and Aquaculture. Ifremer, Centre de Bretagne, 29280 Plouzané, France

Corresponding author: Aihua Li, Tel: 86-27-68780053, E-mail address: liaihua@ihb.ac.cn; Cheng Ji, Tel: 86-512-65881257, E-mail address: cji@suda.edu.cn

* These authors contributed equally to this work.

Figure S1 Rarefaction analysis of the different samples. Rarefaction curves of OTUs clustered at 97% sequence identity across different samples; H.1-4 stand for the intestinal contents of healthy fish, and I.1-7, for the intestinal contents of diseased fish.

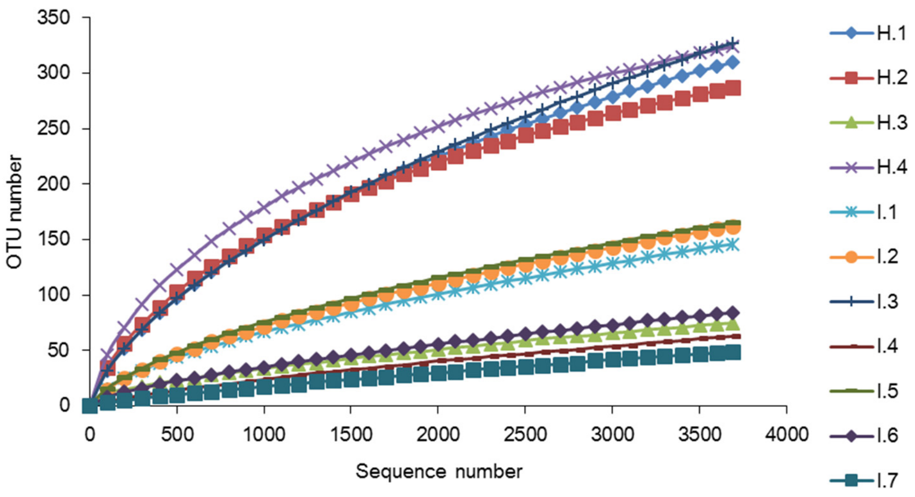

Figure S2 The Venn diagram shows the numbers of OTUs that were shared and unique in the gut communities (97% sequence identity); Healthy and Diseased represent the intestinal samples of healthy and diseased fish.

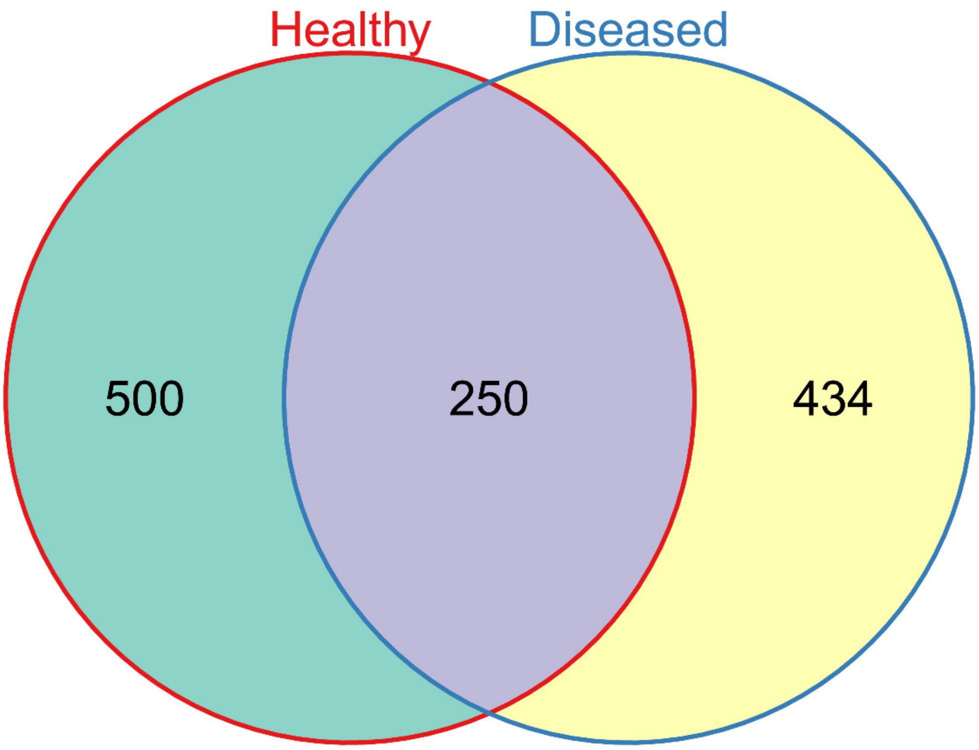

Figure S3 Phylogenetic relationships between OTUs assigned to *Aeromonas* and related bacterium based on 16S rRNA gene sequences. The branching pattern was generated by the neighbour-joining method. Bootstrap was replicated 1,000 times; Bar, 0.05 nuceotide substitutions per site. GeneBank accession numbers are given in parentheses.

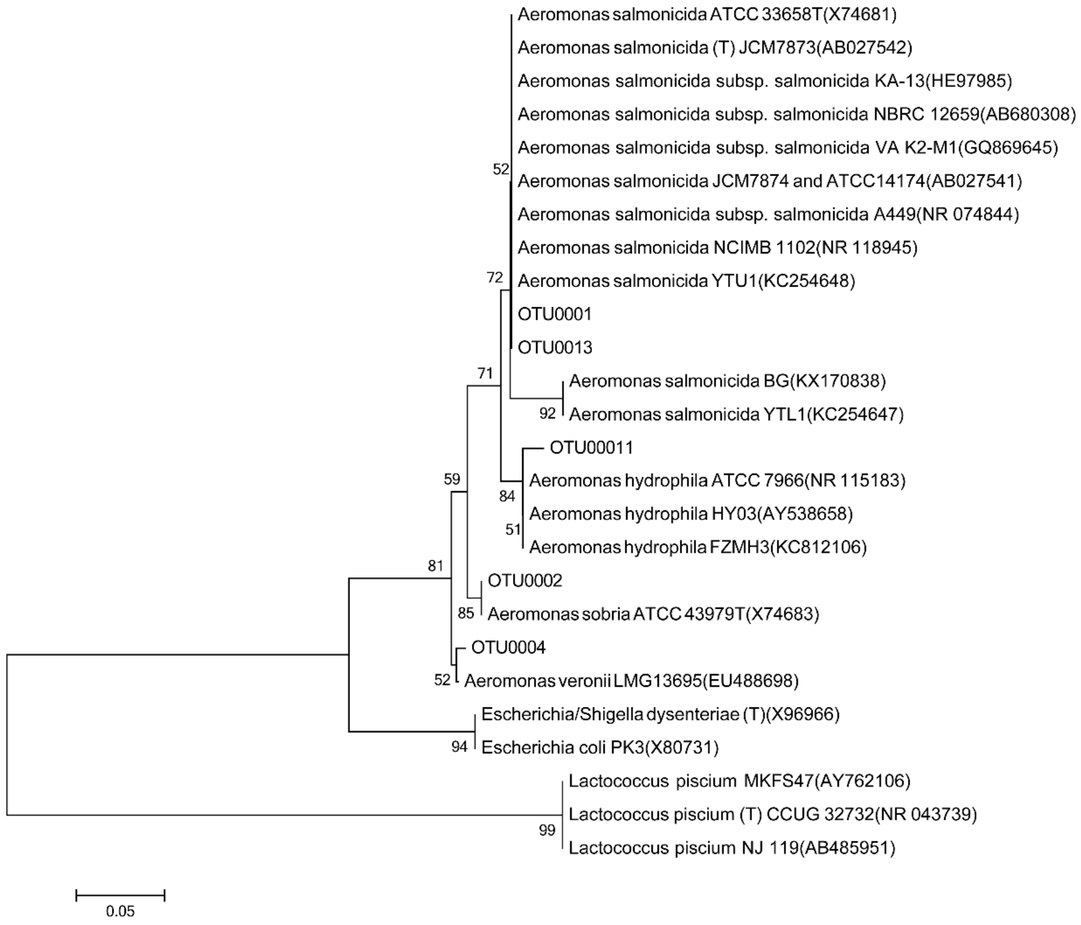

Table S1 Shared OTUs that have relative abundances over 0.5% in at least one group in intestinal microbial communities of *C. guichenoti.*

OUT ID	Taxonomic rank		Shared reads (%)	
	Phylum	Lowest annotated taxon	Healthy	Diseased
OTU0001	Proteobacteria	*Aeromonas*_genus	5.21	75.58
OTU0002	Proteobacteria	*Aeromonas*_genus	15.24	3.62
OTU0003	Tenericutes	*Mycoplasma*_genus	11.50	3.47

OTU0004	Proteobacteria	*Aeromonas*_genus	11.56	0.58
OTU0005	Actinobacteria	unclassified_Actinomycetales_order	13.40	2.04
OTU0006	Proteobacteria	*Escherichia_Shigella*_genus	2.19	0.78
OTU0007	Proteobacteria	*Acinetobacter*_genus	2.14	0.51
OTU0008	Proteobacteria	*Iodobacter*_genus	1.91	0.01
OTU0009	Proteobacteria	*Pseudomonas*_genus	1.55	0.21
OTU0010	Proteobacteria	*Shewanella*_genus	1.00	0.29
OTU0011	Proteobacteria	*Aeromonas*_genus	0.70	0.19
OTU0012	Bacteroidetes	*Flavobacterium*_genus	1.30	0.29
OTU0013	Proteobacteria	*Aeromonas*_genus	0.03	0.82
OTU0014	Fusobacteria	*Cetobacterium*_genus	0.03	0.86
OTU0015	Proteobacteria	unclassified_Neisseriaceae_family	1.15	0.00
OTU0016	unclassified_bacteria	unclassified_bacteria	0.10	0.54
OTU0017	Verrucomicrobia	*Rubritalea*_genus	0.57	0.29
OTU0018	Proteobacteria	*Brevundimonas*_genus	1.02	0.26
OTU0019	Gemmatimonadetes	*Gemmatimonas*_genus	1.34	0.00
OTU0020	Firmicutes	*Anoxybacillus*_genus	0.99	0.43
OTU0021	Proteobacteria	*Buttiauxella*_genus	0.75	0.26
OTU0024	Spirochaetes	*Brevinema*_genus	0.71	0.00
OTU0025	Actinobacteria	unclassified_Pseudonocardiaceae_family	0.51	0.17
OTU0026	Actinobacteria	*Microbacterium*_genus	0.64	0.14
OTU0028	Proteobacteria	*Sphingomonas*_genus	0.62	0.09

Table S2 Classification of the five most abundant unique OTUs in the intestinal samples of healthy fishes, listed from most to least abundant. OTUs were identified using 97% cutoffs.

OUT ID	Taxonomic rank		Shared reads (%)
	Phylum	Lowest annotated taxon	Healthy
OTU0052	Bacteroidetes	*Bacteroides*_genus	0.23
OTU0079	Bacteroidetes	unclassified_Bacteroidetes_phylum	0.16
OTU0078	Bacteroidetes	unclassified_Porphyromonadaceae_family	0.12
OTU0117	Proteobacteria	*Rubellimicrobium*_genus	0.11
OTU0151	Proteobacteria	*Perlucidibaca*_genus	0.11

Table S3 Classification of the five most abundant unique OTUs in the intestinal samples of healthy fishes, listed from most to least abundant. OTUs were identified using 97% cutoffs.

OUT ID	Taxonomic rank		Shared reads (%)
	Phylum	Lowest annotated taxon	Diseased
OTU0035	Proteobacteria	*Gemmiger*_genus	0.16
OTU0072	Cyanobacteria_Chloroplast	GpI_genus	0.10
OTU0071	Firmicutes	*Staphylococcus*_genus	0.07
OTU0083	Proteobacteria	*Aeromonas*_genus	0.07
OTU0085	Proteobacteria	*Glaciecola*_genus	0.07
